# Multiplex PCR to Differentiate Monkeypox Virus Clades

**DOI:** 10.3201/eid3202.250686

**Published:** 2026-02

**Authors:** Christopher T. Williams, Alessandra Romero-Ramirez, Adeleye Adesola Semiu, Samuel Oluwafunmbi Ifabumuyi, Caitlin Greenland-Bews, Susan Gould, Dominic Wooding, Collette Allen, Anushri Somasundaran, Nicodemus Nnabuike Mkpuma, Dorcas Gado, Jolly Amoche Adole, Abdulakeem Eniola Amoo, Abisola Ajoke Adeyemi, Laure Bosquillon de Jarcy, Christine Goffinet, Jake Dunning, Malcolm G. Semple, The International Severe Acute Respiratory, Emerging Infection Consortium Investigators,, Esto Bahizire, Afolabi Akinpelu, Thomas E. Fletcher, Ana I. Cubas-Atienzar, Cristina Leggio, Adeyinka Adedeji, Adesuyi A. Omoare, Thomas Edwards

**Affiliations:** Liverpool School of Tropical Medicine, Liverpool, UK (C.T. Williams, A. Romero-Ramirez, C. Greenland-Bews, S. Gould, D. Wooding, A. Somasundaran, L. Bosquillon de Jarcy, C. Goffinet, T.E. Fletcher, A. Cubas-Atienzar, T. Edwards); Nigeria Centre for Disease Control and Prevention, Abuja, Nigeria (A.A. Semiu, S.O. Ifabumuyi, A. Akinpelu, A.A. Omoare); UK Health Security Agency, Porton Down–Salisbury, UK (C. Allen, C. Leggio); National Veterinary Research Institute, Vom, Nigeria (N.N. Mkpuma, D. Gado, J.A. Adole, A. Adedeji, T. Edwards); Nigeria Centre for Disease Control and Prevention Central Public Health Laboratory, Yaba, Nigeria (A.E. Amoo, A.A. Adeyemi); Charité–Universitätsmedizin Berlin, Berlin, Germany (L. Bosquillon de Jarcy); Berlin Institute of Health, Berlin (L. Bosquillon de Jarcy); University of Oxford, Oxford, UK (J. Dunning); Infectious Diseases Department, Royal Free London NHS Foundation Trust, London, UK (J. Dunning); University of Liverpool, Liverpool (M.G. Semple); Catholic University of Bukavu, Bugabo Campus, Kinshasa, Democratic Republic of the Congo (E. Bahizire)

**Keywords:** MPXV, monkeypox virus, mpox, viruses, PCR, diagnostics, zoonoses, United Kingdom, Nigeria

## Abstract

We designed a multiplex quantitative PCR to differentiate monkeypox virus clades. For clinical samples collected in the United Kingdom and Nigeria, sensitivity was 78% (95% CI 67.67%–86.14%) and specificity 94% (95% CI 80.84%–99.30%); for samples with cycle thresholds <35, sensitivity was 98% (95% CI 91.72%–99.96%) and specificity 94% (95% CI 80.84%–99.30%).

Mpox is a zoonotic viral disease caused by monkeypox virus (MPXV). There are 2 MPXV clades; clade I is historically associated with a higher disease severity and case-fatality ratio compared with clade II (*1*). Clade II is subdivided into IIa and IIb. Lineage B.1 emerged from IIb as the dominant MPXV lineage in 2022 (*2*), with marked human-to-human transmission linked to sexual activity (*3*,*4*) but lower mortality rates (*5*). Clade I is subdivided into Ia and Ib; Ib emerged in the Democratic Republic of the Congo in 2023 (6). Similar to lineage B.1, the clade Ib outbreak resulted in decreased mortality with sustained human-to-human transmission (*6*,*7*). An ≈1 kb deletion occurs in the OPG032 gene in clade Ib (*6*). This segment is the target of the US Centers for Disease Control and Prevention (CDC) clade I PCR (*8*).

Diagnosis of mpox relies on PCR testing performed on lesion swab specimens (*9*). Because the clinical manifestations of mpox are similar between clades, clade identification typically requires sequencing, which is time-consuming, expensive, and difficult to implement in low- and middle-income countries. We designed a multiplex quantitative PCR to differentiate monkeypox virus clades.

## The Study

We used DNA extracted from cultured lineage B.1 MPXV (European Virus Archive Global, https://www.european-virus-archive.com; strain no. Slovenia_MPXV-1_2022, clade hMPXV-1, lineage B.1), and 47 clinical samples from the International Severe Acute Respiratory and Emerging Infection Consortium clinical characterization protocol study (ethics approval no. REC 13/SC/0149). We propagated viral isolates in Vero E6 cells and extracted DNA by using the QIAamp 96 Virus QIAcube-HT Kit (QIAGEN, https://www.qiagen.com) on a QIAcube-HT (QIAGEN), following manufacturer instructions. Samples collected in 2018 (n = 11) were from persons with suspected West Africa travel–associated mpox, and samples from 2022 (n = 36) were from suspected United Kingdom mpox cases. According to the reference CDC mpox PCR (*10*), 32 samples were MPXV-positive. We made minor adjustments to the CDC PCR by reducing the reaction volume and not performing the RNase P assay. All confirmed UK cases from 2018 were clade IIb from West Africa, whereas the UK Health Security Agency sequencing data revealed that 99% of 2022 mpox cases were lineage B.1 (*11*).

We also tested 54 MPXV-positive lesion samples from routine mpox diagnostic surveillance at the National Reference Laboratory (Abuja, Nigeria) of the Nigerian Centre for Disease Control and Prevention (NCDC; approval granted by the Research Governance Unit). Samples were collected in 2017 (n = 7), 2018 (n = 11), 2019 (n = 10), 2021 (n = 10), 2023 (n = 12), and 2024 (n = 4). We used 20 varicella zoster virus PCR-positive samples (PCR-negative for mpox) as negatives. We extracted DNA by using QIAmp DNA mini kits (QIAGEN), following manufacturer instructions. We retrospectively analyzed serial dilutions of DNA from stocks of clade Ia (hMpxV/DRC-INRB/22MPX0422C/2023, 2023-WHO-LS-008) and Ib (hMpxV/DRC-INRB/24MPX0203V/2024, 2024-WHO-LS-003) MPXV to determine limits of detection.

We identified clade-specific mutations by using Nextstrain (genome NC_063383.1; https://nextstrain.org/mpox/all-clades). We downloaded sequences for each clade and lineage from GenBank and aligned through ClustalW by using MEGA 11 (https://www.megasoftware.net). We manually designed the probes to contain mutations in the middle. We designed the primers by using PrimerQuest (Integrated DNA Technologies, https://eu.idtdna.com). The clade I probe targeted the F3L gene (mutation sites T46417C, G46421A, A46427C, C46435T), and the lineage B.1 probe targeted the OPG109 gene (mutation site C84587T). An assay on the OPG210 gene (mutation sites G183695A, C183696T) distinguishes clade IIb from non-IIb; clade IIb contains the F3L and OPG210 gene mutations, whereas clade IIa and clade I do not. The clade Ib assay targets the ≈1 kb deletion (Δ19,128–20,270) in the OPG032 gene (Table 1)

**Table 1:** Assay interpretation of multiplex PCR to differentiate monkeypox virus clades from the United Kingdom and Nigeria*

Result	Probe				
	Clade I (T46417C, G46421A, A46427C, C46435T)	Clade Ib (Δ19,128–20,270)	Non-IIb (G183695, C183696)	Clade IIb (G183695A, C183696T)	Lineage B.1 (C84587T)
Clade Ia	Positive	Negative	Positive	Negative	Negative
Clade Ib	Positive	Positive	Positive	Negative	Negative
Clade IIa	Negative	Negative	Positive	Negative	Negative
Clade IIb	Negative	Negative	Negative	Positive	Negative
Lineage B.1	Negative	Negative	Negative	Positive	Positive

*Values in parentheses are the sites of nucleotide mutations each probe was assigned to.

We used TaqPath-Fast mix (Thermo Fisher Scientific, https://www.thermofisher.com), primers and probes (Appendix), nuclease-free water, and 2.5 µl of DNA for quantitative PCR (qPCR) reactions. To improve specificity for lineage B.1, we designed a blocker oligo identical to the B.1 probe but without the mutation and with a 5′ end phosphate instead of a fluorophore. We conducted experiments on a Quantstudio 5 (Thermo Fisher Scientific) by using the thermal profile 95°C for 20 seconds, followed by 40 cycles of 95°C for 1 second and 60°C for 10 seconds. We used a positivity cutoff cycle threshold (Ct) of 38, with thresholds set at 10% of the maximum fluorescence of the positive control. We used synthetic double-stranded DNA containing target amplicons (Twist Bioscience, https://www.twistbioscience.com) or DNA from viral stocks as positive controls.

We created standard curves of clade I, IIb, and non-IIb DNA controls in triplicate from 1 × 10^4^ to 1 copies/µL (Figure). We quantified DNA extracted from lineage B.1 and clade Ia and Ib viral stocks from the DNA controls to make standard curves. We conducted all analysis with the finalized multiplex. We conducted 20 replicates at 10 copies/µL and 1 copy/µL for each target; all replicates at 10 copies/µL successfully amplified.

**Figure:**
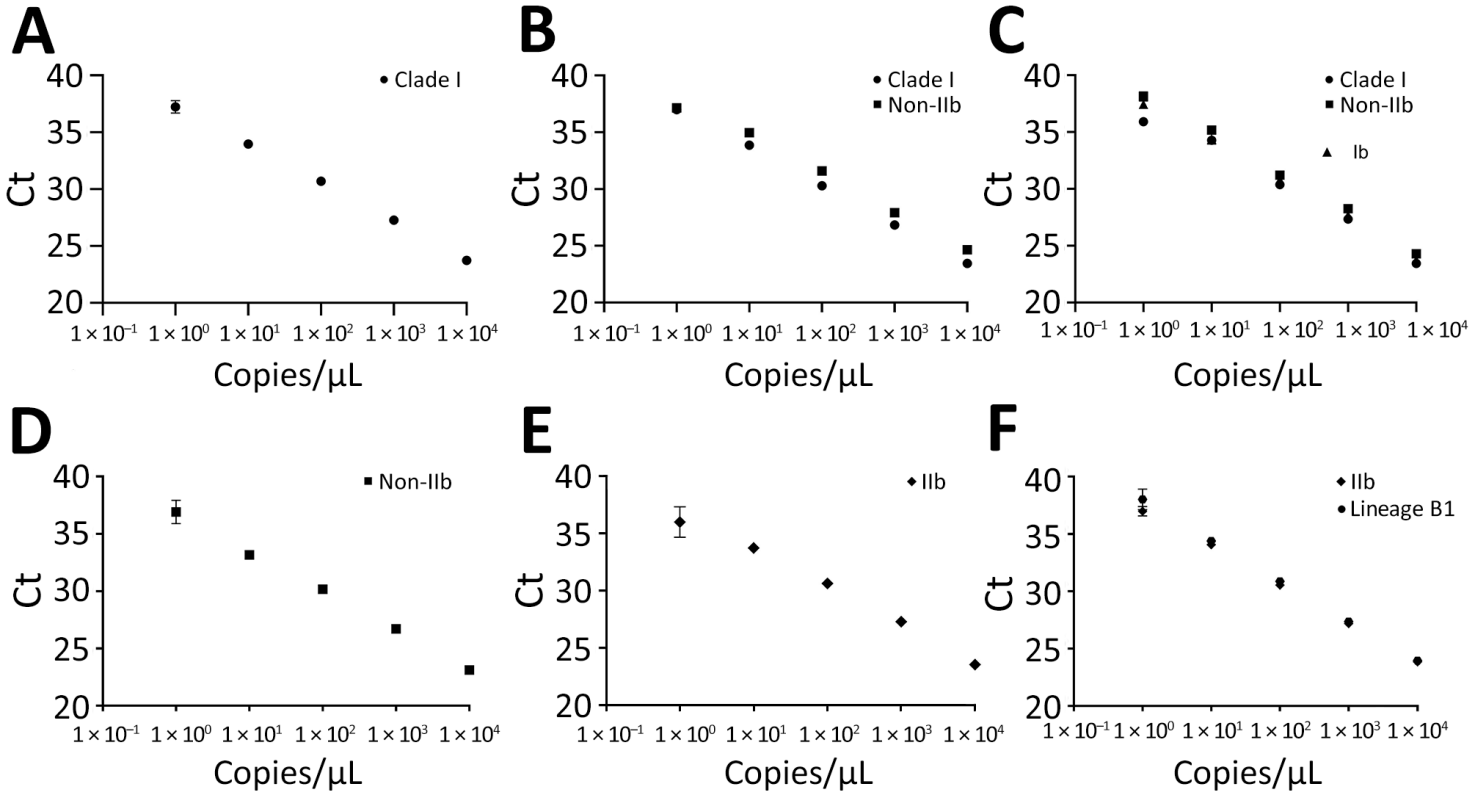
Standard curves of each monkeypox virus PCR target for multiplex PCR to differentiate monkeypox virus clades. A) Synthetic DNA containing clade I amplicon (R^2^ 0.997, efficiency 98.1%). B) DNA extracted from stock of clade Ia virus (clade I curve: R^2^ 0.998, efficiency 96.4%; non-IIb curve: R^2^ 0.992, efficiency 103.394%). C) DNA extracted from stock of clade Ib virus (clade I curve: R^2^ 0.986, efficiency 98.858%; non-IIb curve: R^2^ 0.996, efficiency 93.9%; Ib curve: R^2^ 0.996, efficiency 95.067%). D) Synthetic DNA containing non-IIb amplicon (R^2^ 0.994, efficiency 97%). E) Synthetic DNA containing IIb amplicon (R^2^ 0.98, efficiency 109.2%). F) DNA extracted from stock of lineage B.1 virus (IIb curve: R^2^ 0.999, efficiency 101.4%; lineage B.1 curve: R^2^ 0.996, efficiency 96.6%). Ct, cycle threshold, R^2^, coefficient of determination.

In the UK evaluation, our assay had 97% (95% CI 83.78%–99.92%) sensitivity and 87% (95% CI 59.54%–98.34%) specificity compared with the CDC mpox PCR (Table 2). We tested discrepant samples by using the Sansure Monkeypox Virus Kit (Sansure-Biotech, https://www.sansureglobal.com). The 2 false positives and 1 false negative were positive according to Sansure, suggesting 2 false negative and 1 true positive result by using the CDC PCR. Our assay was 100% specific and 80% sensitive for detecting lineage B.1. The 5 false negatives had Cts > 34.

**Table 2:** Evaluation results of multiplex PCR to differentiate monkeypox virus clades from the United Kingdom and Nigeria*

Reference PCR and country	Result	True positive	True negative	False positive	False negative	Sensitivity, %	Specificity, %
CDC assay, United Kingdom	Total	31	13	2	1	97 (83.78–99.92)	87 (59.54–98.34)
	Total Ct <35	31	13	2	0	100 (88.78–100.00)	87 (59.54–98.34)
	Clade I	0	45	0	0	NA	100 (92.13–100.00)
	IIa	0	45	0	0	NA	100 (92.13–100.00)
	IIb	31	13	2	1	97 (83.78–99.92)	87 (59.54–98.34)
	Lineage B.1	20	22	0	5	80 (59.30–93.17)	100 (84.56–100.00)
CDC with Sansure, United Kingdom†	Total	33	13	0	1	97 (84.67–99.93)	100 (75.29–100.00)
CDC Assay, Nigeria	Total	36	20	0	18	67 (52.53–78.91)	100 (83.16–100.00)
	Total Ct <35	32	20	0	1	97 (84.24–99.92)	100 (83.16–100.00)
CDC assay; combined, United Kingdom and Nigeria	Total	67	33	2	19	78 (67.67–86.14)	94 (80.84–99.30)
	Total Ct <35	64	33	2	1	98 (91.72–99.96)	94 (80.84–99.30)

*Numbers in parentheses are 95% CIs. Ct, cycle threshold; NA, not applicable. †Discrepant samples were tested by using the Sansure Monkeypox virus Kit (Sansure-Biotech, https://www.sansureglobal.com).

In the NCDC evaluation, our assay showed sensitivity of 67% (95% CI 52.53%–78.91%) and specificity of 100% (95% CI 83.16%–100.00%) compared with the CDC assay (Table 2). Of the available samples, 20% (n = 11) had a Ct >35; typically, Ct values from lesion swab specimens in the acute phase are lower (*12*), and samples with Ct >35 are predicted to have no or very little infectivity (*13*). In samples with Ct <35, we observed 97% sensitivity (95% CI 84.24%–99.92%) and 100% specificity (95% CI 83.16%–100.00%). All samples were confirmed to be clade IIb. One sample had a Ct of 25.59 for IIb and 36.77 for B.1; this difference discounted a true B.1 positive and was classified as a false positive, giving a 98% specificity for this target.

## Conclusions

Sensitivity for our assay was high in samples with a reference qPCR Ct <35 but lower with higher Ct values, supporting assay use for reflex testing samples with a Ct <35. Clade I and IIa clinical samples were unavailable. Although the other assays compared were created on the basis of multiple single nucleotide polymorphisms, the lineage B.1 assay is based on a single mutation. If this single mutation were to naturally occur in nonlineage B.1 strains, it could compromise the assay. Mutations will be monitored on Nextstrain. We assigned clinical samples to clades on the basis of date and location of collection after failed attempts to generate full genome assemblies after sequencing. Although this process is suboptimal, 99% of MPXV sequenced in the United Kingdom in 2022 by UK Health Security Agency was lineage B.1; the 2018 samples were imported cases from Nigeria, and only clade IIb is known to have circulated in Nigeria during our collection dates (*4*). Other assays can distinguish between clades; a qPCR that detects clade I, clade II, clade IIb, and lineage B.1 was previously published (*14*) but requires 2 tubes per sample and does not include clade Ib. Another assay detects clade Ib by using singleplex assays (*15*).

Rapid identification of MPXV clades is vital with outbreaks occurring attributed to different clades. Clade and lineage identification is necessary because of differences in disease severity and epidemiologic tracking, particularly for new outbreaks. Although sequencing is the standard diagnostic practice, PCR remains necessary when access to sequencing is limited or extra throughput is required.

Appendix Additional information about multiplex PCR to differentiate monkeypox virus clades.
