# Supplementary material for: Multiplex PCR to Differentiate Monkeypox Virus Clades
Source: Emerg Infect Dis. 2026 Feb;32(2):250–4. doi: 10.3201/eid3202.250686 (PMC12928233; doi:10.3201/eid3202.250686)
Supplement: Appendix — Additional information about multiplex PCR to differentiate monkeypox virus clades. [file 25-0686-Techapp-s1.pdf]

Article DOI: https://doi.org/10.3201/eid3202.250686

EID cannot ensure accessibility for supplementary materials supplied by authors. Readers who have difficulty accessing supplementary content should contact the authors for assistance.

Multiplex PCR to Differentiate Monkeypox Virus Clades

Appendix

Supplementary data; DNA controls

Clade I Control (312 bp), F3L gene (T46417C, G46421A, A46427C, C46435T) KP849470.1

CCCCACTGATTCAATACGAAAAGACCAATCTCTCCTAGTTATTTGGCAGTACT
CATTAATAACGGTGACAGGGTTAACACCTTTCCAATAAATAATTTTTTTAACCGGAA
TAACATCATCAAAAGACTTATTATCCTCTCTCATTGATTTTTCGCGGGATACATCATC
TATTATAGCATCAGCATCAGAATCTGTAGGCCGTGTATCAGCATCCATTGTCGTAGA
CCAACGAGGAGGAGTATCGTCGGAACTGTACACCATAGTACTACGTTGAAGATCAT
ACAGAGCTTTATTAACTTCTCGCTTCTCCAT

Clade IIb Control (451 bp), OPG210 gene (G183695A, C183696T) NC_06383.1

CCTCCTCTCCGTAATCCACTTCCTCAACATGATGATTATTCTCCTCCACAAGT
ACACAGACCTCCACCACTTCCTCCTAAACCAGTCCAAAATTCGCCACAACTTCCCCC
TAGACCAGTAGGTCAATTACTACCTCCTCCTATAGATCAACCAGATAAAGGATTTAG
TAAGTTTGTATCACCTAGACGGTGTAGAAGAGCAAGCTCTGGAGTCATATGTGGTAT
GATACAATCAAAACCAAACGATGATACCTATTCACTTCTTCAACGATCAAAAATTGA
ACCAGAATATGTGGAGGTTGGTAATGGTATACCCAAGAACAATGTTCCTGTAATAG
GTAATAAACATAGTAAAAAATATACATCGACGATGTCAAAAATATCAACAAAATTT
GATAAATCTACGGCATTTGGAGCAGCAATGTTACTAACTGGTCAGCAGGCCATTAGC
C

Non-IIb control (451 bp), OPG210 gene, WT (G183695, C183696) KP849470.1

CCTCCTCTCCGTAATCCACTTCCTCAACATGATGATTATTCTCCTCCACAAGT ACACAGACCTCCACCACTTCCTCCTAAACCAGTCCAAAATTCGCCACAACTTCCCCC TAGACCAGTAGGTCAATTACTACCTCCTCCTATAGATCAACCAGATAAAGGATTTAG TAAGTTTGTATCACCTAGACGGTGTAGAAGAGCAAGCTCTGGAGTCATATGTGGTAT GATACAATCAAAACCAAACGATGATACCTATTCACTTCTTCAACGGCCAAAAATTGA ACCAGAATATGTGGAGGTTGGTAATGGTATACCCAAGAACAATGTTCCTGTAATAG GTAATAAACATAGTAAAAAATATACATCGACGATGTCAAAAATATCAACAAAATTT GATAAATCTACGGCATTTGGAGCAGCAATGTTACTAACTGGTCAGCAGGCCATTAGC C

Appendix Table 1. Primers and probes used in the assay*

Oligo name	Mod5'	Sequence	Mod3'	Concentration
Clade I For		TCTGTAGGCCGTGTATCAGC		400nM
Clade I Rev		AAGCTCTGTATGATCTTCAACGT		400nM
Clade I Probe	Cy5	AGGAGTATCGTCGGAACTGTACACCATAGT	BHQ	200nM
Clade Ib For		TCCGTTTGATATAGGATGTGGAC		500nM
Clade Ib Rev		ATATTTGAAACACGGCACTTCG		500nM
Clade Ib Probe	Texas Red	CACGTGGGTGGATAATGCGCCTGAATAT	BHQ 2	250nM
IIb/non-IIb For		TCAAAACCAAACGATGATACCTATTCACT		450nM
IIb/non-IIb Rev		CCAACCTCCACATATTCTGGTTCA		450nM
IIb Probe	FAM	TTCAACGATCAAAAAT	MGB	100nM
non-IIb Probe	VIC	AACGGCCAAAAAT	MGB	100nM
Lineage B.1 For		TGGAGAAC[G]TCAACGTGTATC		400nM
Lineage B.1 Rev		GATCACAAG[G]CTGGTACAGA		400nM
Lineage B.1 Probe	NED	CATACGATATCTTTGGTATTGA	MGB	200nM
Lineage B.1 PHO blocker	PHO	CATACGATATCTTCGGTATTGA		1mM

*For, forward; rev, reverse

Appendix Table 2. Results from UK evaluation*

Sample Code	Year	Assigned clade	CDC Ct (CO Ct 40)	CDC Result	Mpox assay (CO Ct 38)				Interpretation
					B1	IIb	non-IIb	clade I	
Sample 1	2018	IIb	21.25	Positive	Negative	20.41	Negative	Negative	IIb, non-B1
Sample 2	2018	IIb	31.08	Positive	Negative	30.34	Negative	Negative	IIb, non-B1
Sample 3	2018	IIb	32.45	Positive	Negative	30.97	Negative	Negative	IIb, non-B1
Sample 4	2018	IIb	29.67	Positive	39.89	28.37	Negative	Negative	IIb, non-B1
Sample 5	2018	IIb	28.31	Positive	Negative	27.46	Negative	Negative	IIb, non-B1
Sample 6	2018	IIb	Negative	Negative	Negative	Negative	Negative	Negative	Negative
Sample 7	2018	IIb	Negative	Negative	Negative	Negative	Negative	Negative	Negative
Sample 8	2018	IIb	39.69	Positive	Negative	35.95	Negative	Negative	IIb, non-B1
Sample 9	2018	IIb	37.08	Positive	Negative	34.36	Negative	Negative	IIb, non-B1
Sample 10	2018	IIb	25.63	Positive	Negative	24.37	Negative	Negative	IIb, non-B1
Sample 11	2018	IIb	Negative	Negative	Negative	35.88	Negative	Negative	IIb, non-B1
Sample 12	2022	Lineage B.1	30.82	Positive	30.15	28.53	Negative	Negative	IIb, B1
Sample 13	2022	Lineage B.1	39.72	Positive	Negative	34.79	Negative	Negative	IIb, non-B1
Sample 14	2022	Lineage B.1	33.40	Positive	32.45	31.11	Negative	Negative	IIb, B1
Sample 15	2022	Lineage B.1	32.61	Positive	31.17	29.91	Negative	Negative	IIb, B1
Sample 16	2022	Lineage B.1	39.70	Positive	Negative	36.50	Negative	Negative	IIb, non-B1
Sample 17	2022	Lineage B.1	26.67	Positive	35.41	24.85	Negative	Negative	IIb, B1
Sample 18	2022	Lineage B.1	31.54	Positive	29.94	28.75	Negative	Negative	IIb, B1
Sample 19	2022	Lineage B.1	21.02	Positive	19.98	19.11	Negative	Negative	IIb, B1
Sample 20	2022	Lineage B.1	33.06	Positive	32.58	30.50	Negative	Negative	IIb, B1
Sample 21	2022	Lineage B.1	33.31	Positive	32.29	31.27	Negative	Negative	IIb, B1
Sample 22	2022	Lineage B.1	27.70	Positive	26.45	25.54	Negative	Negative	IIb, B1
Sample 23	2022	Lineage B.1	36.61	Positive	36.62	35.19	Negative	Negative	IIb, B1
Sample 24	2022	Lineage B.1	33.30	Positive	32.91	31.01	Negative	Negative	IIb positive, B1 positive

Sample Code	Year	Assigned clade	CDC Ct (CO Ct 40)	CDC Result	Mpox assay (CO Ct 38)				Interpretation
					B1	IIb	non-IIb	clade I	
Sample 25	2022	Lineage B.1	Negative	Negative	Negative	38.18	Negative	Negative	IIb positive, B1 negative
Sample 26	2022	Lineage B.1	Negative	Negative	Negative	Negative	Negative	Negative	Negative
Sample 27	2022	Lineage B.1	27.09	Positive	26.74	24.92	Negative	Negative	IIb positive, B1 positive
Sample 28	2022	Lineage B.1	Negative	Negative	Negative	Negative	Negative	Negative	Negative
Sample 29	2022	Lineage B.1	28.71	Positive	36.61	27.72	Negative	Negative	IIb positive, B1 positive
Sample 30	2022	Lineage B.1	28.26	Positive	27.15	25.80	Negative	Negative	IIb positive, B1 positive
Sample 31	2022	Lineage B.1	Negative	Negative	Negative	39.73	Negative	Negative	Negative
Sample 32	2022	Lineage B.1	Negative	Negative	Negative	Negative	Negative	Negative	Negative
Sample 33	2022	Lineage B.1	24.91	Positive	24.14	22.76	Negative	Negative	IIb positive, B1 positive
Sample 34	2022	Lineage B.1	Negative	Negative	Negative	Negative	Negative	Negative	Negative
Sample 35	2022	Lineage B.1	Negative	Negative	Negative	Negative	Negative	Negative	Negative
Sample 36	2022	Lineage B.1	30.97	Positive	29.17	27.27	Negative	Negative	IIb positive, B1 positive
Sample 37	2022	Lineage B.1	27.65	Positive	26.63	25.09	Negative	Negative	IIb positive, B1 positive
Sample 38	2022	Lineage B.1	Negative	Negative	Negative	Negative	Negative	Negative	Negative
Sample 39	2022	Lineage B.1	Negative	Negative	Negative	Negative	Negative	Negative	Negative
Sample 40	2022	Lineage B.1	Negative	Negative	Negative	Negative	Negative	Negative	Negative
Sample 41	2022	Lineage B.1	Negative	Negative	Negative	Negative	Negative	Negative	Negative
Sample 42	2022	Lineage B.1	29.33	Positive	25.35	24.74	Negative	Negative	IIb positive, B1 positive
Sample 43	2022	Lineage B.1	28.47	Positive	28.16	26.11	Negative	Negative	IIb positive, B1 positive
Sample 44	2022	Lineage B.1	35.39	Positive	Negative	34.86	Negative	Negative	IIb positive, B1 negative
Sample 45	2022	Lineage B.1	Negative	Negative	Negative	37.90	Negative	Negative	IIb positive, B1 negative
Sample 46	2022	Lineage B.1	26.37	Positive	25.01	23.43	Negative	Negative	IIb positive, B1 positive
Sample 47	2022	Lineage B.1	39.82	Positive	Negative	Negative	Negative	Negative	Negative

*CDC, Centers for Disease Control and Prevention; Ct, cycle threshold

Appendix Table 3. Results from Nigeria evaluation

Sample	Year	NCDC, CDC Assay Ct CO 40	NCDC Result	Mpox real-time PCR Ct 38 CO				Interpretation
				Clade IIb	Lineage B.1	non-IIb	Clade I	
NCDC mpox 1	2017	32.64	Positive	38.03	Negative	Negative	Negative	Negative
NCDC mpox 2	2017	18.84	Positive	20.25	Negative	Negative	Negative	IIb
NCDC mpox 3	2017	23.80	Positive	24.31	Negative	Negative	Negative	IIb
NCDC mpox 4	2017	26.21	Positive	30.25	Negative	Negative	Negative	IIb
NCDC mpox 5	2017	21.38	Positive	26.22	Negative	Negative	Negative	IIb
NCDC mpox 6	2018	34.77	Positive	37.87	Negative	Negative	Negative	IIb
NCDC mpox 7	2018	22.64	Positive	24.79	Negative	Negative	Negative	IIb
NCDC mpox 8	2018	33.88	Positive	34.70	Negative	Negative	Negative	IIb
NCDC mpox 9	2018	19.64	Positive	19.34	Negative	Negative	Negative	IIb
NCDC mpox 10	2018	26.79	Positive	27.90	Negative	Negative	Negative	IIb
NCDC mpox 11	2018	38.58	Positive	Negative	Negative	Negative	Negative	Negative
NCDC mpox 12	2018	23.07	Positive	24.05	Negative	Negative	Negative	IIb
NCDC mpox 13	2018	26.72	Positive	30.56	Negative	Negative	Negative	IIb
NCDC mpox 14	2018	24.56	Positive	29.88	Negative	Negative	Negative	IIb
NCDC mpox 15	2018	23.65	Positive	30.07	Negative	Negative	Negative	IIb
NCDC mpox 16	2018	38.29	Positive	38.47	Negative	Negative	Negative	Negative
NCDC mpox 17	2018	23.23	Positive	25.00	Negative	Negative	Negative	IIb
NCDC mpox 18	2018	26.70	Positive	27.95	Negative	Negative	Negative	IIb
NCDC mpox 19	2019	23.05	Positive	26.48	Negative	Negative	Negative	IIb
NCDC mpox 20	2019	27.43	Positive	31.60	Negative	Negative	Negative	IIb
NCDC mpox 21	2019	22.54	Positive	24.09	Negative	Negative	Negative	IIb
NCDC mpox 22	2019	36.44	Positive	Negative	Negative	Negative	Negative	Negative
NCDC mpox 23	2019	20.97	Positive	20.98	Negative	Negative	Negative	IIb
NCDC mpox 24	2019	36.05	Positive	Negative	Negative	Negative	Negative	Negative
NCDC mpox 25	2019	35.16	Positive	37.38	Negative	Negative	Negative	IIb

Sample	Year	NCDC, CDC Assay Ct CO 40	NCDC Result	Mpox real-time PCR Ct 38 CO				Interpretation
				Clade IIb	Lineage B.1	non-IIb	Clade I	
NCDC mpox 1	2017	32.64	Positive	38.03	Negative	Negative	Negative	Negative
NCDC mpox 26	2019	35.32	Positive	Negative	Negative	Negative	Negative	Negative
NCDC mpox 27	2019	22.20	Positive	22.80	Negative	Negative	Negative	IIb
NCDC mpox 28	2019	25.07	Positive	25.86	Negative	Negative	Negative	IIb
NCDC mpox 29	2021	35.27	Positive	35.95	Negative	Negative	Negative	IIb
NCDC mpox 30	2021	21.22	Positive	21.91	Negative	Negative	Negative	IIb
NCDC mpox 31	2021	24.02	Positive	23.27	Negative	Negative	Negative	IIb
NCDC mpox 32	2021	22.39	Positive	21.13	Negative	Negative	Negative	IIb
NCDC mpox 33	2021	24.34	Positive	26.93	39.79	Negative	Negative	IIb
NCDC mpox 34	2021	23.47	Positive	22.76	Negative	Negative	Negative	IIb
NCDC mpox 35	2021	20.82	Positive	20.69	Negative	Negative	Negative	IIb
NCDC mpox 36	2021	26.01	Positive	25.59	36.77	Negative	Negative	IIb, B.1
NCDC mpox 37	2021	25.96	Positive	27.23	Negative	Negative	Negative	IIb
NCDC mpox 38	2021	24.35	Positive	23.95	Negative	Negative	Negative	IIb
NCDC mpox 39	2023	38.23	Positive	38.06	Negative	Negative	Negative	Negative
NCDC mpox 40	2023	38.69	Positive	38.76	Negative	Negative	Negative	Negative
NCDC mpox 41	2023	36.76	Positive	39.01	Negative	Negative	Negative	Negative
NCDC mpox 42	2023	36.60	Positive	37.88	Negative	Negative	Negative	IIb
NCDC mpox 43	2023	38.86	Positive	38.32	Negative	Negative	Negative	Negative
NCDC mpox 44	2023	38.89	Positive	Negative	Negative	Negative	Negative	Negative
NCDC mpox 45	2023	39.32	Positive	Negative	Negative	Negative	Negative	Negative
NCDC mpox 46	2023	20.21	Positive	20.79	Negative	Negative	Negative	IIb
NCDC mpox 47	2023	38.63	Positive	38.56	Negative	Negative	Negative	Negative
NCDC mpox 48	2023	35.60	Positive	36.53	Negative	Negative	Negative	IIb
NCDC mpox 49	2023	38.68	Positive	Negative	Negative	Negative	Negative	Negative
NCDC mpox 50	2023	25.19	Positive	24.62	Negative	Negative	Negative	IIb
NCDC mpox 51	2024	37.23	Positive	Negative	Negative	Negative	Negative	Negative
NCDC mpox 52	2024	38.58	Positive	Negative	Negative	Negative	Negative	Negative
NCDC mpox 53	2024	36.89	Positive	Negative	Negative	Negative	Negative	Negative
NCDC mpox 54	2024	37.76	Positive	Negative	Negative	Negative	Negative	Negative

*CDC, Centers for Disease Control and Prevention; Ct, cycle threshold, NCDC, Nigerian Centre for Disease Control and Prevention.
